# Comprehensive analysis of the biological function and immune infiltration of SLC38A2 in gastric cancer

**DOI:** 10.1186/s12876-023-02689-4

**Published:** 2023-03-14

**Authors:** Liang Zhu, Zhengguang Wang, Wenxiu Han, Aman Xu

**Affiliations:** Department of General Surgery, The First Affiliated Hospital of Anhui Medical University, No. 218 Jixi Road, Hefei, Anhui 230001 People’s Republic of China

**Keywords:** Solute carrier family 38 member 2, Gastric cancer, Proliferation, Prognosis, Immune infiltration

## Abstract

**Background:**

Solute carrier family 38 member 2 (SLC38A2) has previously been reported to participate in carcinogenesis. However, its expression and function in gastric cancer (GC) remain unclear. The present study aimed to investigate the role of SLC38A2 in GC.

**Methods:**

The prognostic value and expression of SLC38A2 in GC was analyzed by combining bioinformatics and experimental analyses. Colony formation, Cell Counting Kit-8, wound healing, Transwell and tumor formation assays were performed to assess the biological function of SLC38A2. The cBioPortal, GeneMANIA and LinkedOmics databases were mined to determine the underlying regulatory mechanisms of SLC38A2. The role of SLC38A2 in tumor immune infiltration was explored using the TIMER database.

**Results:**

Our results demonstrated that SLC38A2 was upregulated and was correlated with a poor prognosis in GC patients. SLC38A2 downregulation significantly inhibited the proliferation, invasion and migration of GC cells. Abnormal genetic alteration and epigenetic regulation may contribute to the upregulation of SLC38A2 expression levels in GC. The results of enrichment analysis demonstrated that SLC38A2 was associated with ‘hippo signaling’ and ‘ubiquitinyl hydrolase activity’. The results also indicated that SLC38A2 may be a key factor in GC immune infiltration and M2 macrophage polarization.

**Conclusion:**

Overall, these data identified that SLC38A2 may serve as a potential prognostic biomarker and therapeutic target in GC.

**Supplementary Information:**

The online version contains supplementary material available at 10.1186/s12876-023-02689-4.

## Background

Gastric cancer (GC) is a common type of cancer with a mortality rate that was ranked the fifth highest globally in 2018 [1]. Although modern treatments have improved GC prognoses, the overall survival rate of patients with GC remains low [2]. The poor prognosis of patients with GC is related to a lack of therapeutic alternatives, particularly in chemotherapy-resistant patients [3]. Previous studies have highlighted that tumor-infiltrating lymphocytes play an important role in regulating the efficiency of immunotherapy and chemotherapy [4–6]. Therefore, novel immune-related therapeutic targets, as well as information regarding their immunological interactions with GC, need to be investigated.

Amino acids are essential for cell protein synthesis, proliferation, metabolism, signal transduction and stress responses [7]. Specific cell membrane transporters can accumulate amino acids to support various cellular biochemical signaling pathways. Among them, solute carrier family 38 member (SLC38A) 2 is a secondary active transporter powered by the Na^+^ electrochemical gradient and is responsible for the transport of neutral amino acids, especially glutamine [8]. Moreover, glutamine is the main nutrient used by cancer cells in energy metabolism [9], which indicates the importance of SLC38A2 in cancer metabolism. Previous studies have reported that abnormal SLC38A2 expression can induce cancer-like metabolic characteristics and promote breast and pancreatic cancer [10, 11]. However, the biological function and underlying mechanisms of SLC38A2 in GC remain unclear.

The aim of the present study was to explore the expression level, prognostic value, potential regulatory mechanism and protein interaction network of SLC38A2 in GC. Furthermore, enrichment analysis of SLC38A2 in GC was performed, and the correlation between SLC38A2 and tumor-infiltrating immune cells was analyzed. The findings of the current research may provide a potential molecular treatment target and a valuable immune infiltration factor for GC.

## Materials and methods

### Clinical tissue specimens

Tissue samples were collected from patients with GC at the First Affiliated Hospital of Anhui Medical University (Hefei, China). Patients were first consulted before all data were collected with their consent. The present study was approved by the Academic Committee of Anhui Medical University (approval no. 20,150,232).

### Cell culture

The human gastric mucosal cell line GES1 (control cell line) and gastric cancer cell lines MKN45, AGS, MKN1 and HGC27 were purchased from The Cell Bank of Type Culture Collection of The Chinese Academy of Sciences. Cells were cultured in RPMI-1640 medium or DMEM (Biological Industries), supplemented with 10% FBS (Biological Industries). Before experiments were performed, short tandem repeat assays were used to authenticate all cell lines (Shanghai Biowing Applied Biotechnology Co., Ltd.).

### Stable transfection

Human SLC38A2 short hairpin (sh)-RNA and empty vector lentiviral particles were constructed by Qingke Co., Ltd. The sh1-SLC38A2 target sequence was 5’- CCTCCAATCCTCTGGCTATTT-3’ and the sh2-SLC38A2 target sequence was 5’- CCTGAACAATGAATTCCCATT-3’. Lentiviruses were transfected into the GC MKN45 and HGC27 cell lines according to the manufacturer’s protocol. The stably transfected cells were selected using 2 µg/ml puromycin (Beijing Solarbio Science & Technology Co., Ltd.) for at least 2 weeks. The efficiency of transfection was assessed via western blotting.

### Reverse transcription-quantitative PCR (RT-qPCR)

TRIzol® (Beyotime Institute of Biotechnology) was used to extract RNA from cells according to the manufacturer’s protocol. A cDNA Reverse Transcription Kit (Vazyme Biotech Co., Ltd.) was used to synthesize the first-strand of cDNA. Subsequently, qPCR was performed using SYBR Green PCR Master Mix (Vazyme Biotech Co., Ltd.). GAPDH was used as an internal reference gene. qPCR primers are presented in Table S1. The 2^−ΔΔCq^ method was used to determine relative mRNA expression levels.

### Western blotting

Western blotting was used to determine protein expression levels. Briefly, cells were lysed at 4˚C for 30 min using RIPA cell lysis buffer (Beyotime Institute of Biotechnology) containing 1 mmol/l PMSF and then centrifuged at 13,800 x g for 10 min at 4˚C. A BCA kit (Beyotime Institute of Biotechnology) was used to determine the protein concentration. Total protein (20 µg protein/lane) from each sample was separated via SDS-PAGE on a 10% gel. Separated protein was subsequently transferred to 0.45 μm PVDF membranes (MilliporeSigma). Membranes were then incubated in 5% nonfat milk for 1 h at room temperature and then incubated with the primary anti-SLC38A2 antibody (1:1,000; cat. no. sc-166,366; Santa Cruz Biotechnology, Inc.) overnight at 4˚C. After washing with TBS with Tween-20, the membrane was incubated for 2 h at room temperature with the secondary antibody (1:5,000; cat. no. ab6728; Abcam). Chemiluminescence was used to visualize the membranes.

### Immunohistochemistry (IHC)

IHC was performed using a specific kit (Boster Biological Technology) according to the manufacturer’s protocol. The percentage of positive tumor cells and the intensity of their staining were used to estimate SLC38A2 protein expression levels. The final IHC scores were determined according to the immunostaining intensity (negative, 0; weak, 1; moderate, 2; and strong, 3) and the percentage of positively stained cells (negative, 0; 0–25%, 1; 26–50%, 2; 51–75%, 3; and 76–100%, 4). Scores from 0 to 7 represented low SLC38A2 expression levels and scores from 8 to 12 were regarded as high expression levels. The staining procedure and results were independently evaluated by two pathologists.

### Colony formation assay

Cell proliferation was assessed using a colony formation assay. Into 6-well microplates ~ 200 cells/well were seeded and incubated at 37˚C in 5% CO_2_. After 2 weeks, the cells were fixed using 4% paraformaldehyde (Biomiky) for 30 min and stained with crystal violet for 30 min at room temperature. Subsequently, the cells were imaged using a microscope.

### Cell Counting Kit-8 (CCK-8) assay

Cell proliferation was also assessed using a CCK-8 assay kit (Beyotime Institute of Biotechnology) according to the manufacturer’s protocol. After plating the cells with different treatments in 96-well plates, CCK-8 reagent was added to each well for 4 h in a 37˚C incubator according to the manufacturer’s instructions. Cell proliferation was determined by assessing the absorbance at 450 nm using a microplate reader.

### Migration assay

The wound healing assay was used to assess cell migratory ability. Cells (~ 5.0 × 10^5^ cells/well) were seeded into 6-well plates and cultured to achieve > 80% confluence. A 200 µl pipette tip was then used to form a scratch in each well. After washing twice with PBS, serum-free medium was added. Images were taken with a light microscope (magnification, x40; Olympus Corporation) at 0 and 48 h after the scratch had been formed.

### Invasion assay

The Transwell assay was used to assess cell invasive ability. Cells (~ 1.0 × 10^5^) were seeded into the upper chamber, which had been precoated with Matrigel and heated to 37˚C. The lower chamber was filled with 750 µl DMEM supplemented with 10% FBS. After 24 h of incubation at 37˚C in 5% CO_2_, the invading cells were fixed with 4% paraformaldehyde for 20 min and stained with crystal violet for 20 min at room temperature. Cells were imaged using a light microscope (magnification, x100; Olympus Corporation).

### *In vivo* experiments

A total of 12 healthy, male BALB/c nude mice (age, 4–6 weeks) were purchased from the Experimental Animal Center of Anhui Medical University. The sh-SLC38A2 and corresponding sh-negative control transfected MKN45/HGC27 cells were injected subcutaneously into the ipsilateral armpit of nude mice. The mice were raised in a specific pathogen-free environment at 18–22 °C, with a humidity of 50–60% under a 12-h light/dark cycle and fed sterile food. All mice had free access to food and water. Tumor sizes were measured every 4 days. The mice were sacrificed by cervical dislocation after 16 days, and tumors were excised to assess their volume (when the heart stopped completely, the mouse was determined to be dead). No mice died accidentally during the experiment. The Ethics Committee of the First Affiliated Hospital of Anhui Medical University approved the animal experiments performed in the present study (approval no. 20,150,234). Also, all the procedures were complied with Directive 2010/63/EU in Europe.

### Public database analysis

The UALCAN database was used to analyze the Cancer Genome Atlas (TCGA) data to identify the expression level of SLC38A2 in GC tumors and normal tissues (Group cutoff for separating patients into SLC38A2 high and low expression groups was set as median) [12]. The Kaplan-Meier Plotter database was used to assess the prognostic value and levels of SLC38A2 using a Mann-Whitney U-test and the False discovery rate setted at 5% in R software environment [13]. cBioPortal was used to assess SLC38A2 gene copy number variations and mutations (one-way analysis of variance, P-value < 0.0001) [14, 15]. The protein-protein network of SLC38A2 was constructed using the GeneMANIA database [16]. The GeneMANIA algorithm uses an association-by-association approach to derive predictions from a combination of potentially heterogeneous data sources [16]. Moreover, the LinkedOmics [17] database was mined to find SLC38A2 co-expressed genes based on the Spearman’s rank correlation coefficient and the results were displayed using a volcano plot and heatmap. Linear regression between SLC38A2 and its top three positively/negatively associated genes and M2 macrophage cell markers was confirmed using the GEPIA2 database (P-value < 0.05) [18]. Moreover, enrichment analysis using Gene Ontology (GO) annotations and Kyoto Encyclopedia of Genes and Genomes (KEGG) [19] pathways was performed using LinkedOmics (Select tool: Gene Set Enrichment Analysis; Rank Criteria: P-value; Simulations: 500 times) [17]. LinkedOmics was also searched via gene set enrichment analysis (GSEA) to explore microRNA- (miRNAs/miRs) and transcription factor-target enrichment [17]. Furthermore, the Tumor Immune Estimation Resource (TIMER) database was used [20] to analyze correlations between immune cell infiltration, including CD8 + T cells, CD4 + T cells and B cells, and SLC38A2 expression. The correlation coefficients were determined using Spearman’s rank correlation coefficient test (P-value < 0.05).

### Statistical analysis

All *in vitro* and *in vivo* data are based on three independent experiments. A Student’s t-test was used to analyze significant differences between two groups. Statistical differences among more than two groups were analyzed using one-way ANOVA followed by a Tukey’s post hoc test. The Kaplan-Meier method was used to estimate survival analyses. All statistical analyses were performed using Prism 8.0 (GraphPad Software, Inc.) and SPSS 21.0 software (IBM Corp). P < 0.05 was considered to indicate a statistically significant difference.

## Results

### SLC38A2 is upregulated and is associated with a poor prognosis in GC patients

**Fig. 1:**
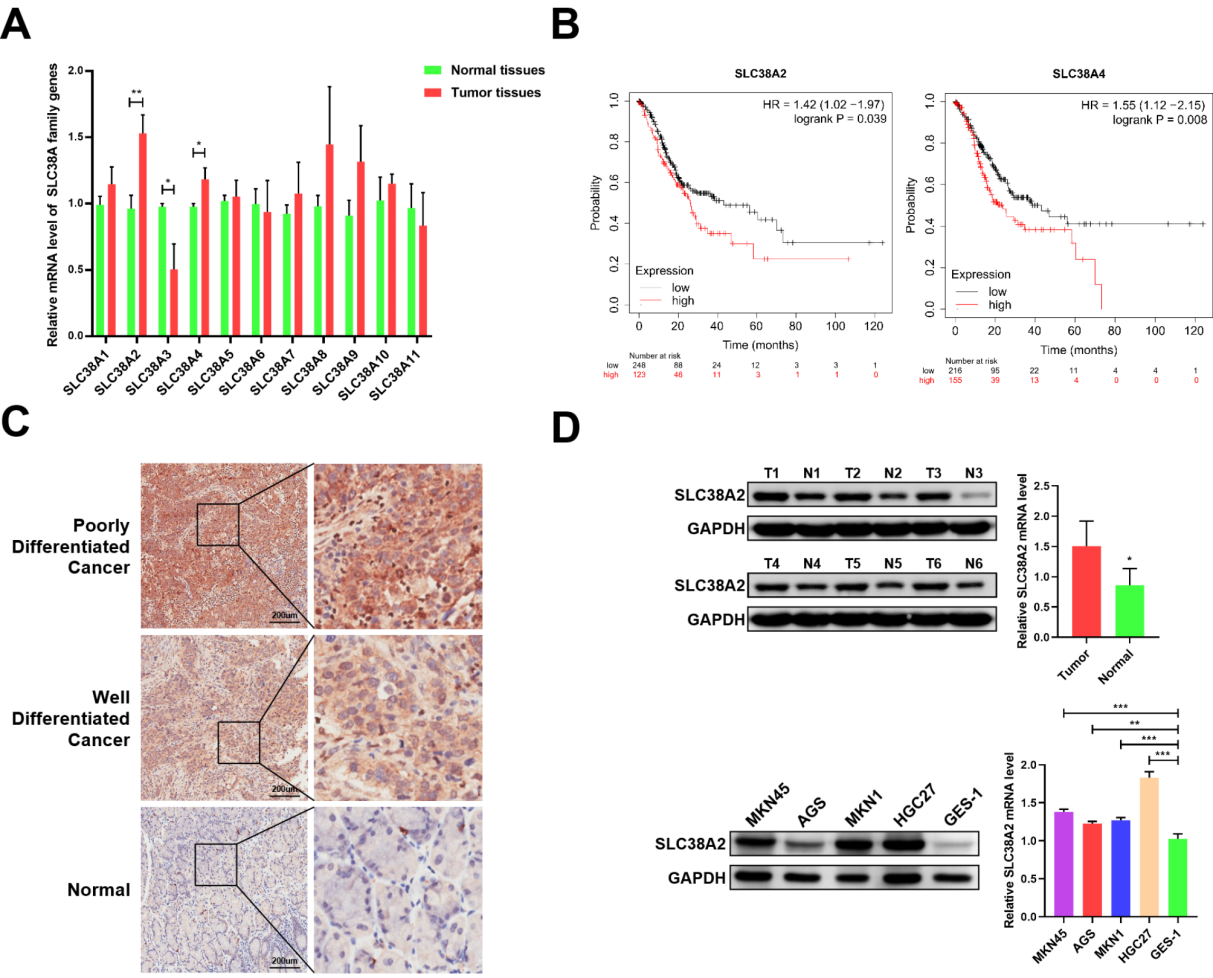
**SLC38A2 is overexpressed and predicts poor prognosis of patients in GC.** (A) The mRNA expression level of various SLC39A family members between tumor and normal tissues in GC (*P < 0.05, **P < 0.01). (B) The prognostic value of SLC38A2 and SLC38A4 in GC patients (HR: hazard ratio). (C) Representative IHC staining images of SLC39A2 expression in normal, well and poorly differentiated cancer tissues. (D) SLC38A2 expression level in GC tumor tissues and corresponding normal tissues (*P < 0.05); (E) SLC38A2 expression level in human gastric mucosal cells (GES1) and GC cell lines (*P < 0.05, **P < 0.01, ***P < 0.001)

By analyzing TCGA data in the GEPIA database, we found that the expression levels of multiple SLC38A family members (including SLC38A2, SLC38A4, SLC38A5, SLC38A6, SLC38A7, SLC38A8, SLC38A9, and SLC38A10) were significantly upregulated in GC tumor tissues (Fig. S1). Subsequently, the results of RT-qPCR experiments demonstrated that the mRNA expression levels of SLC38A2, SLC38A3 and SLC38A4 were significantly overexpressed in GC tumor tissues (Fig. 1A). Further prognostic analyses indicated that SLC38A2 and SLC38A4 overexpression were associated with poor prognosis in GC patients (Fig. 1B). Considering the biological role of SLC38A2 in GC remains unclear, SLC38A2 was selected as the primary molecular target for subsequent experiments. IHC, western blotting and RT-qPCR indicated that expression of SLC38A2 was significantly elevated in GC tissues compared to normal adjacent tissues (Fig. 1C and D). Remarkably, the SLC38A2 expression level has been found to be significantly higher in poorly differentiated than in well differentiated cancer tissues. Furthermore, SLC38A2 protein and mRNA expression levels were higher in MKN45, AGS, MKN1 and HGC27 cell lines than in the GES1 cell line (Fig. 1D).

### SLC38A2 promotes tumor cell proliferation, invasion and migration in GC *in vitro* and *in vivo*

**Fig. 2:**
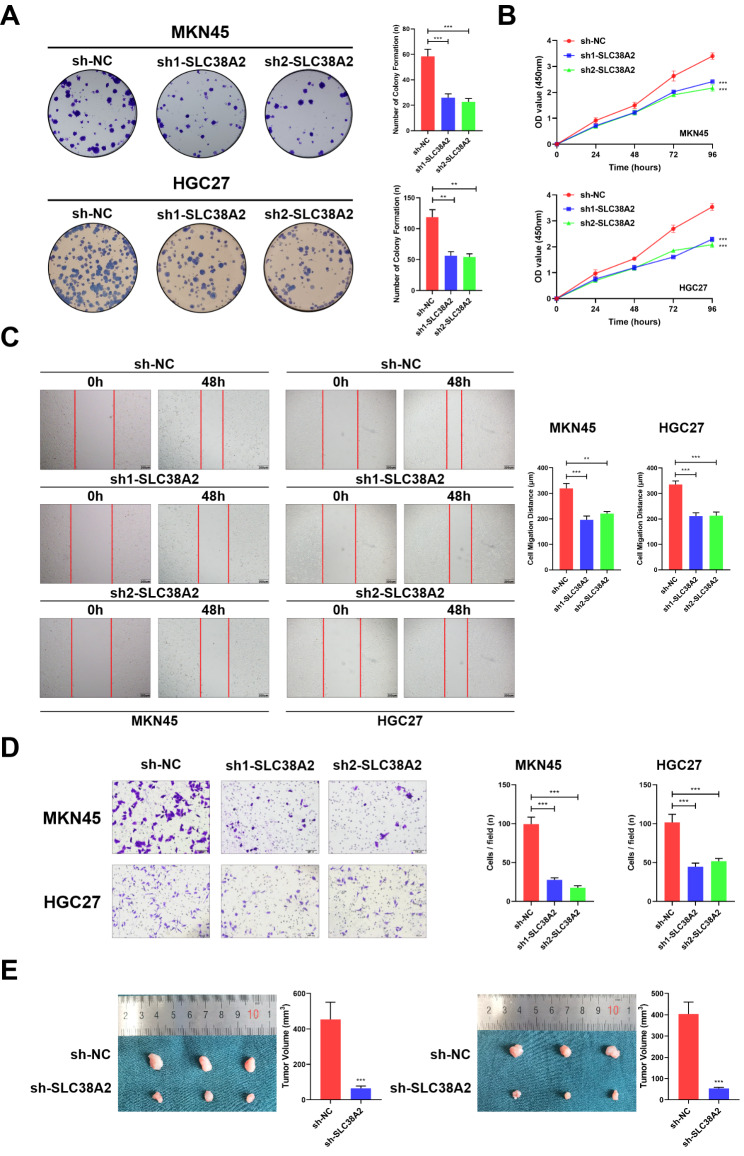
**Tumor-promoting effects of SLC39A2 in GC cells.** (A) Representative images of colony formation induced by sh-NC, sh1- SLC38A2 and sh2- SLC38A2 in MKN45 and HGC27 cell lines (**P < 0.01, ***P < 0.001). (B) CCK8 assay was used to compare cell viability in MKN45 and HGC27 cells with SLC38A2 knockdown (***P < 0.001). (C) A cell wound-healing assay showed that cell motility was decreased after SLC38A2 knockdown in the MKN45 and HGC27 cell lines (**P < 0.01, ***P < 0.001). (D) Cell invasion assays of sh-NC, sh1- SLC38A2 and sh2- SLC38A2 in MKN45 and HGC27 cell lines (***P < 0.001). (E) Representative images of nude mouse xenograft tumors. Statistical analysis of xenograft tumor sizes revealed that tumor growth was markedly inhibited by SLC38A2 silencing (***P < 0.001)

To assess the role of SLC38A2 in GC, two shRNAs (sh1-SLC38A2 and sh2-SLC38A2) were used to silence SLC38A2. Plate colony formation assays showed that knockdown of SLC38A2 significantly reduced cell proliferation (Fig. 2A). The results of CCK-8 analysis also demonstrated that knockdown of SLC38A2 in MKN45 and HGC27 cells significantly reduced cell viability. The OD value of MKN45 cells with knockdown of SLC38A2 increased to an average of 2.42 and 2.17 after 96 h, which was significantly lower than that of the control group of 3.40. The OD value of HGC27 cells with knockdown of SLC38A2 increased to an average of 2.29 and 2.08 after 96 h, which was significantly lower than that of the control group of 3.54 (Fig. 2B). Transwell and wound healing assays were subsequently used to explore its role in cell invasion and migration, respectively. The knockdown of SLC38A2 suppressed GC invasion and migration (Fig. 2C and D). To further validate the effect of SLC38A2 expression *in vivo*, a xenograft tumor model was constructed in nude mice. The results demonstrated that SLC38A2 knockdown significantly reduced tumor induction and formation (Fig. 2E).

### Regulatory network of SLC28A2 in GC

**Fig. 3:**
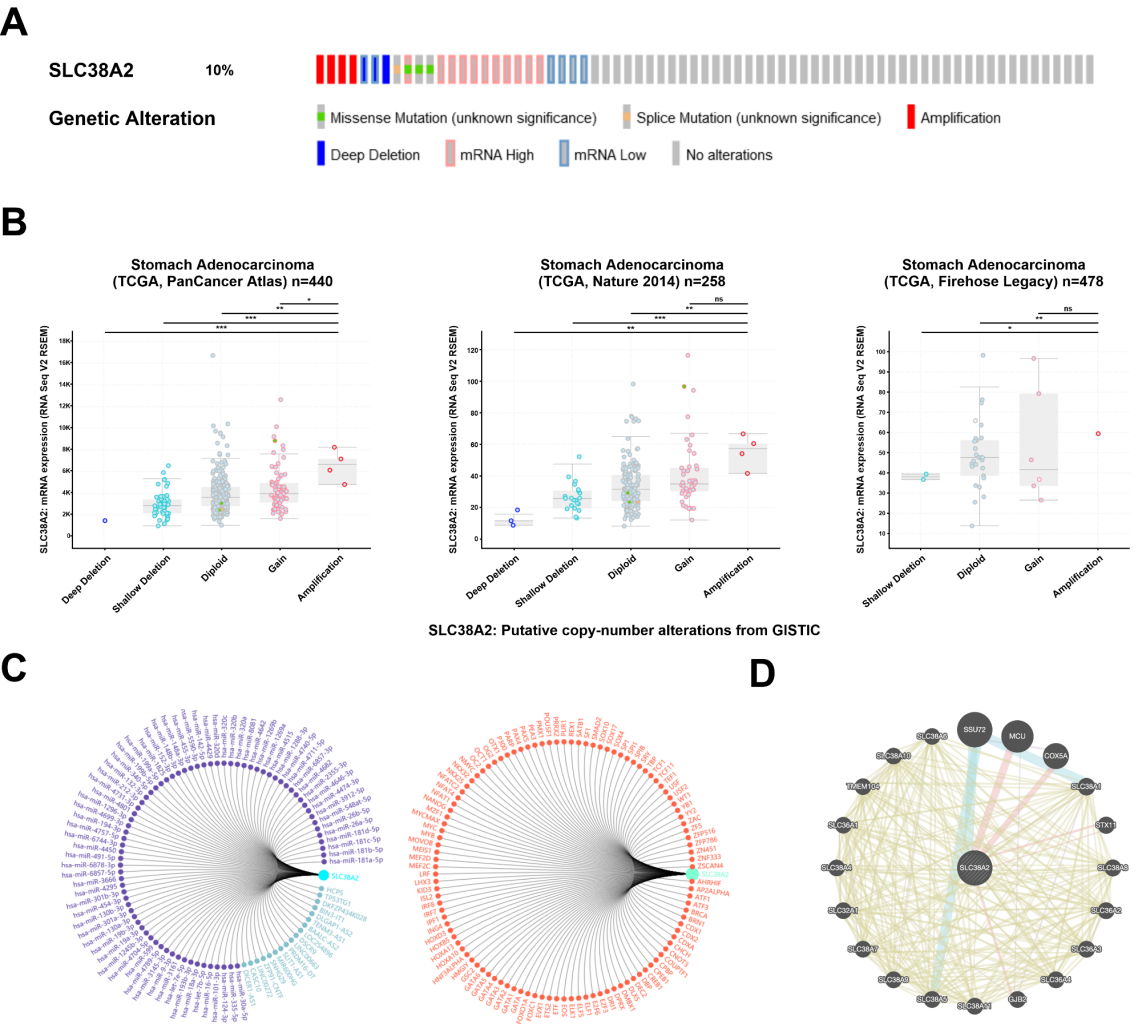
**Regulatory network of SLC38A2 in GC.** (A) SLC38A2 gene alteration in GC is presented as bar diagram; (B) The graph depicts the correlation between SLC38A2 expression and copy number alterations in gastric cancer of TCGA data. (Abbreviations: deep deletions = DD, shallow deletion = SD, diploid = D, gain = G, and amplification = A; NS: not significant, ****P < 0.0001). (C) miRNA and transcription factors network of SLC38A2. (D) PPI network of SLC38A2 in GC by GeneMANIA.

The genetic alteration of SLC38A2 in GC was previously demonstrated via cBioPortal. The results of the present study demonstrated that SLC38A2 was altered in 25 (10%) patients with GC (Fig. 3A). In GC, amplification was significantly correlated with SLC38A2 expression levels in the TCGA PanCancer Atlas, Nature 2014 and Firehose Legacy databases (Fig. 3B). Furthermore, 18 long noncoding RNAs, 74 miRNAs and 109 transcription factors related to SLC38A2 were identified using the Gene-Cloud of Biotechnology Information database (Fig. 3C). Moreover, using the GeneMANIA database, 20 proteins were identified via protein-protein interaction analysis (Fig. 3D). For bioinformatics analysis steps see Fig. S2.

### Enrichment analysis and co-expression profiles of SLC28A2 in GC

**Fig. 4:**
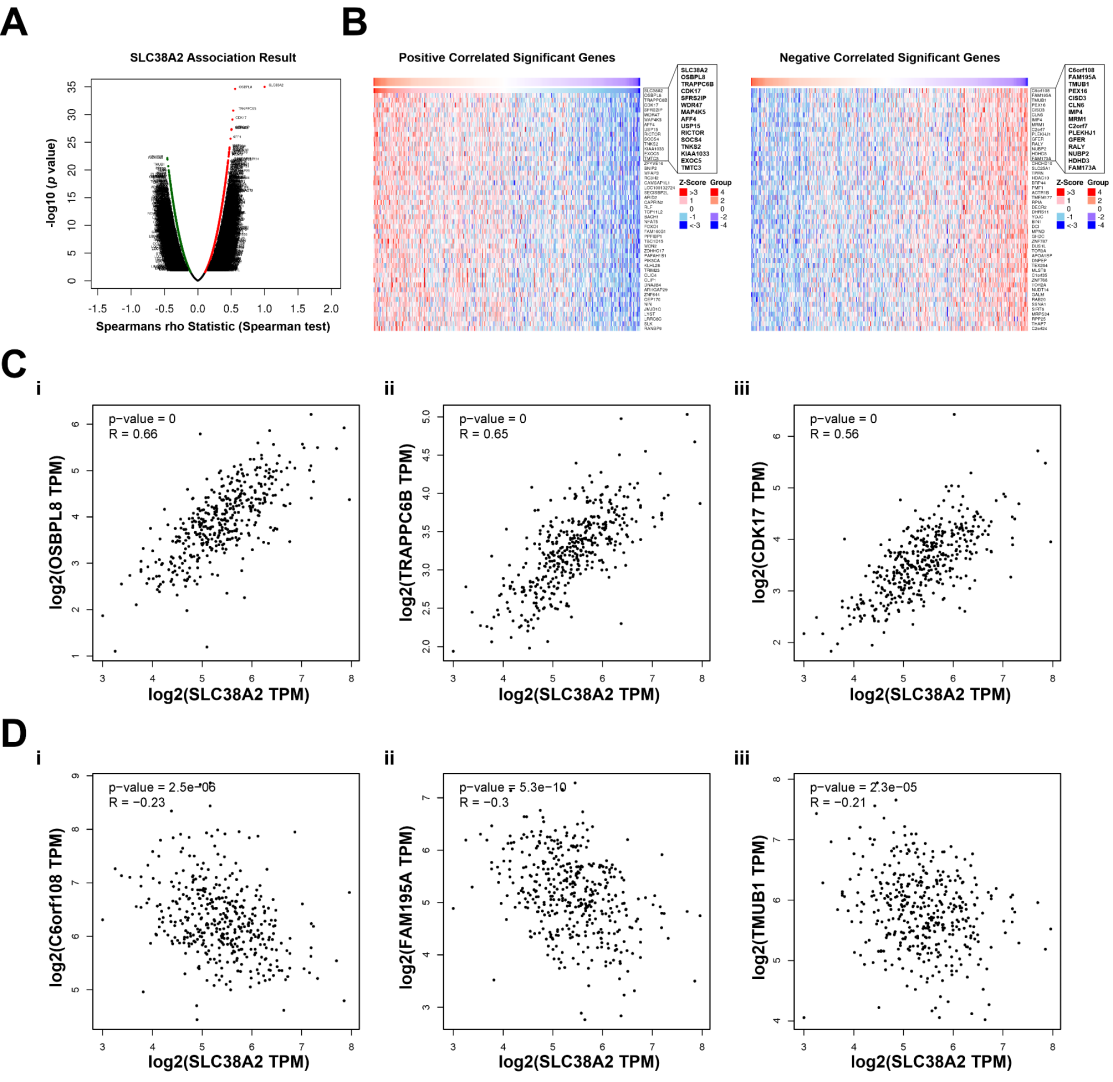
**Analysis of SLC38A2 co-expressed genes in GC.** (A) The volcano plot of SLC38A2 and its correlated genes was analyzed using LinkedOmics; (B) The heatmap of SLC38A2 correlated genes: The top 50 positively correlated significant genes(left-panel); The top 50 negatively correlated significant genes(right-panel). (C) Linear regression relationships between SLC38A2 and its top three positive genes: (i) SLC38A2 and OSBPL8; (ii) SLC38A2 and TRAPPC6B; (iii) SLC38A2 and CDK17 (p-values = 0 means that it is lower than 2.22e-16); (D) Linear regression relationship between SLC38A2 and its top three negative genes: (i) SLC38A2 and c6orf108; (ii) SLC38A2 and FAM195A; (iii) SLC38A2 and TMUB1.

To better understand the role of SLC38A2 in GC development, the LinkedOmics database was used to analyze SLC38A2 co-expressed genes. The results demonstrated that SLC38A2 was positively correlated with 12,767 genes, whereas it was negatively correlated with 7,458 genes in the GC dataset (Fig. 4A). The top 50 genes, which were significantly positively or negatively associated with SLC38A2, were assessed using a heatmap (Fig. 4B) and the precise values are presented in Table S2. The top three genes that were positively associated with SLC38A2 expression were oxysterol binding protein-like 8, trafficking protein particle complex subunit 6B and CDK17, whereas the top three genes negatively associated with SLC38A2 expression were 2’-deoxynucleoside 5’-phosphate N-hydrolase 1, MAPK regulated corepressor interacting protein 2 and transmembrane and ubiquitin-like domain containing 1 (TMUB1). Subsequently, GEPIA2 was used to confirm the relationships between SLC38A2 and these six co-expressed genes (Fig. 4C and D).

**Fig. 5:**
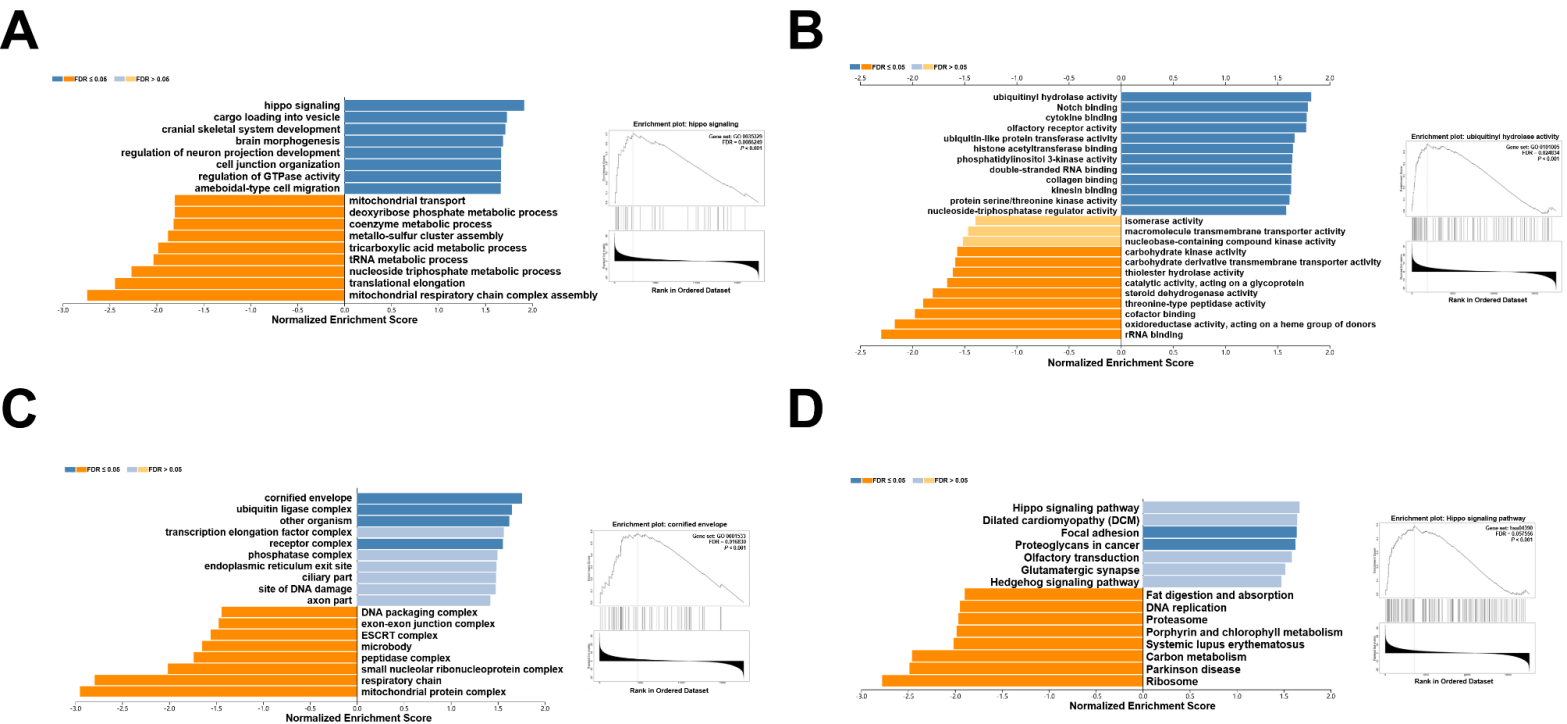
**GO annotation and KEGG pathway analyses by LinkedOmics using GSEA methods.** (A) Bar chart of Biological Process; (B) Bar chart of Molecular Function; (C) Bar chart of Cellular Component; (D) Bar chart of KEGG pathway

LinkedOmics was used to examine GO annotations and KEGG pathways. ‘Hippo signaling’ and ‘cargo loading into vesicle’ were identified as the two most important terms for biological processes (Fig. 5A), whereas ‘ubiquitinyl hydrolase activity’ and ‘notch binding’ were the most abundant molecular function terms (Fig. 5B). The top two cellular component terms were ‘cornified envelope’ and ‘ubiquitin ligase complex’ (Fig. 5C). KEGG pathway analysis demonstrated that the related pathways were ‘Hippo signaling pathway’ and ‘dilated cardiomyopathy’ (Fig. 5D).

Furthermore, miRNAs and transcription factor targets of SLC38A2 in GC were analyzed using LinkedOmics. The top five miRNA targets of SLC38A2 were as follows: (i) TGAATGT (miR-181a, miR-181b, miR-181c and miR-181d); (ii) TTGCACT (miR-130a, miR-301 and miR-130b); (iii) GCACTTT (miR-17-5p, miR-20a, miR-106a, miR-106b, miR-20b and miR-519d); (iv) TAGGTCA (miR-192 and miR-215); and (v) GACTGTT (miR-212 and miR-132) (Table 1). Moreover, transcription factor enrichment analysis revealed that SLC38A2 expression was associated with SOX5_01, V$FOXO4_01 and V$PR_02.

**Table 1:** The miRNAs, and transcription factor-target networks of SLC38A2 in GC.

Enriched category	Gene set	Leading edge number	NES	FDR
**miRNA Target**	TGAATGT,MIR-181 A,MIR-181B,MIR-181 C,MIR-181D	197	2.3326	0
	TTGCACT,MIR-130 A,MIR-301,MIR-130B	157	2.2749	0
	GCACTTT,MIR-17-5P,MIR-20 A,MIR-106 A,MIR-106B,MIR-20B,MIR-519D	221	2.2371	0
	TAGGTCA,MIR-192,MIR-215	19	2.0650	0
	GACTGTT,MIR-212,MIR-132	71	1.9937	0
**Transcription Factor Target**	V$EVI1_06	9	1.9211	0
	KRCTCNNNNMANAGC_UNKNOWN	35	-2.1931	0
	V$SOX5_01	72	1.8762	0.00037417
	TTGTTT_V$FOXO4_01	672	1.7899	0.00066030
	V$PR_02	41	1.7836	0.00070895

### Correlation analysis of SLC38A2, immune infiltration level and representative immune marker genes in GC

**Fig. 6:**
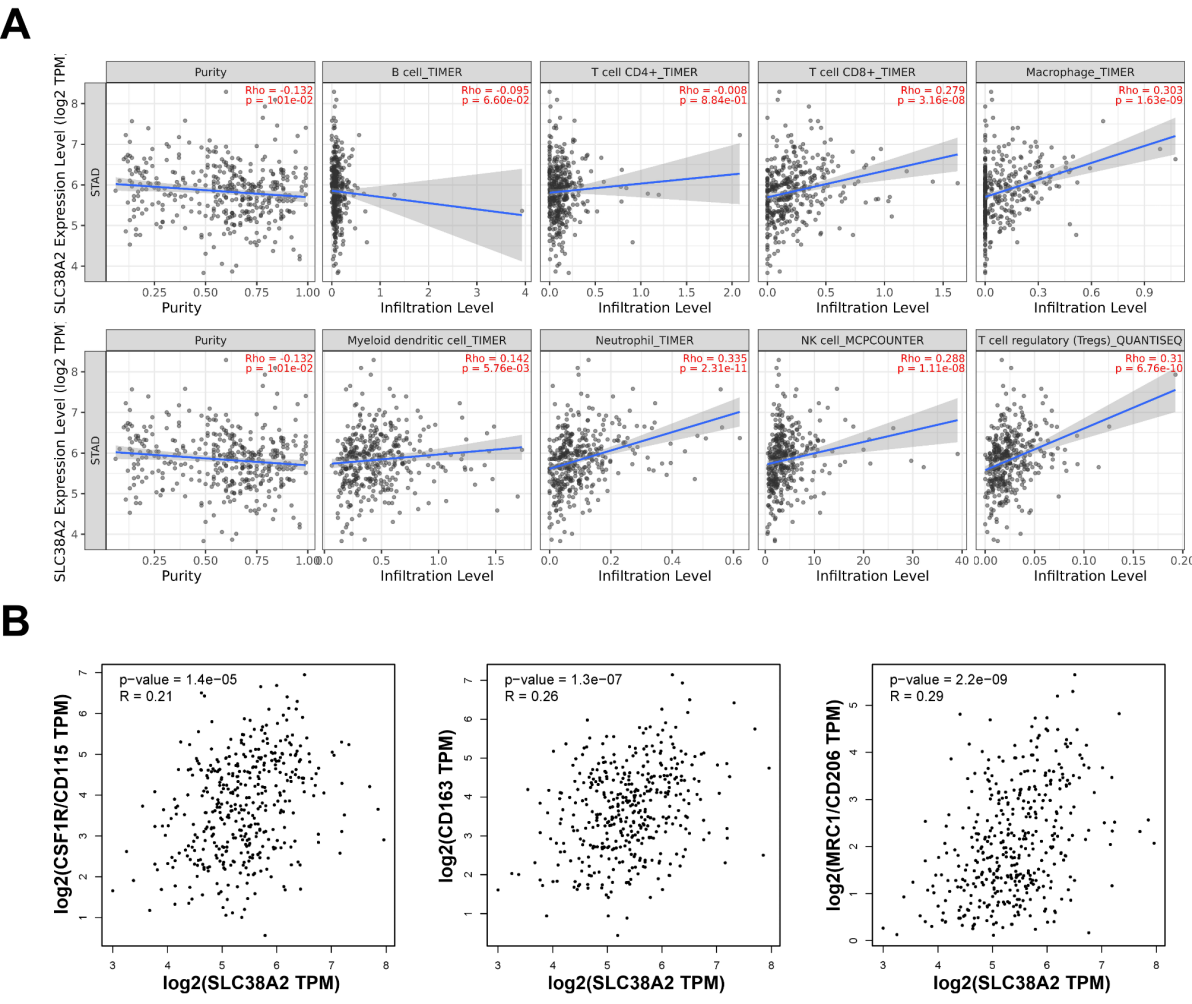
**The correlation between SLC38A2 expression and immune infiltration in GC.** (A) Correlation of SLC38A2 expression with tumor purity and infiltrating levels of B cells, CD8 + T cells, CD4 + T cells, macrophages, neutrophils and dendritic cells in GC; (B) Correlation between SLC38A2 expression and macrophage related gene markers

Cancer occurrence and progression are linked to immune infiltration in the tumor microenvironment. Therefore, the TIMER database was used to investigate the association between SLC38A2 expression and immune infiltration in GC. The results demonstrated that SLC38A2 was significantly correlated with tumor purity in GC (ρ=-0.132; P = 1.01 × 10^− 2^; Fig. 6A). Moreover, SLC38A2 expression was also significantly correlated with the infiltration levels of CD8 + T cells (ρ = 0.279; P = 3.16 × 10^− 8^), macrophages (ρ = 0.303; P = 1.63 × 10^− 9^), myeloid dendritic cells (ρ = 0.142; P = 5.76 × 10^− 3^), neutrophils (ρ = 0.335; P = 2.31 × 10^− 11^), natural killer cells (ρ = 0.288; P = 1.11 × 10^− 8^) and regulatory T cells (Tregs; ρ = 0.31; P = 6.76 × 10^− 10^) (Fig. 6A). The association between SLC38A2 expression and M2 macrophage markers was also examined and the results demonstrated that SLC38A2 expression was significantly associated with colony stimulating factor 1 receptor (ρ = 0.21; P = 1.4 × 10^− 5^), CD163 (ρ = 0.26; P = 1.3 × 10^−^7) and mannose receptor C-type 1 (ρ = 0.29; P = 2.2 × 10^− 9^) (Fig. 6B).

Subsequently, the association between SLC38A2 expression and the status of tumor-infiltrating immune cells, based on the levels of immune marker genes in GC, was investigated. The immune cells analyzed in GC tissues included CD8 + cells, B cells, tumor-associated macrophages (TAMs), neutrophils and dendritic cells. Moreover, different subsets of T cells, including T cells (general), T helper (Th)1, Th2, Th17 and Tregs, were also examined. The association between immune marker genes and SLC38A2 expression with or without tumor purity is presented in Table 2. The results demonstrated that SLC38A2 expression was markedly associated with the markers of specific immune cells as follows: T cells, CD3D (ρ=-0.315; P = 3.37 × 10^− 10^), CD3E (ρ=-0.335; P = 2.15 × 10^− 11^), CD2 (ρ=-0.303; P = 1.72 × 10^− 9^); B cells, CD19 (ρ=-0.218; P = 1.73 × 10^− 5^), CD79A (ρ=-0.268; P = 1.08 × 10^− 7^); TAMs, C-C motif chemokine ligand 2 (ρ=-0.205; P = 5.18 × 10^− 5^), IL10 (ρ=-0.254; P = 5.38 × 10^− 7^); neutrophils, C-C motif chemokine receptor 7 (ρ=-0.292; P = 6.92 × 10^− 9^); dendritic cells, blood dendritic cell antigen-1 (ρ=-0.285; P = 1.63 × 10^− 8^); Th1, T-box expressed in T cells (ρ=-0.254; P = 5.52 × 10^− 7^), STAT4 (ρ=-0.245; P = 1.32 × 10^− 6^), TNF-α (ρ=-0.281; P = 2.62 × 10^− 8^); Tregs, forkhead box P3 (ρ=-0.241; P = 1.94 × 10^− 6^); and T exhaustion cell, granzyme B (ρ=-0.254; P = 5.41 × 10^− 7^). These results suggested that there may be an important association between SLC38A2 expression and GC immune infiltration.

**Table 2:** Correlation analysis between SLC38A2 and related genes and markers of immune cells in STAD in TIMER.

Description	Gene markers	Purity		None	
		Cor	*P*	Cor	*P*
**CD8 + T cell**	CD8A	-0.22	1.54e-05	0.128	1.27e-02
	CD8B	-0.121	1.82e-02	0.042	4.12e-01
**T cell (general)**	CD3D	-0.315	3.37e-10	0.039	4.54e-01
	CD3E	-0.335	2.15e-11	0.06	2.48e-01
	CD2	-0.303	1.72e-09	0.121	1.80e-02
**B cell**	CD19	-0.218	1.73e-05	0.076	1.42e-01
	CD79A	-0.268	1.08e-07	0.029	5.73e-01
**Monocyte**	CD86	-0.286	1.45e-08	0.195	1.36e-04
	CD115 (CSF1R)	-0.208	4.29e-05	0.189	2.20e-04
**TAM**	CCL2	-0.205	5.81e-05	0.065	2.04e-01
	CD68	-0.159	1.87e-03	-0.012	8.13e-01
	IL10	-0.254	5.38e-07	0.269	1.09e-07
**M1 Macrophage**	INOS (NOS2)	-0.094	6.63e-02	-0.062	2.28e-01
	IRF5	-0.111	3.03e-02	0.11	3.25e-02
	COX2(PTGS2)	-0.126	1.40e-02	0.256	4.30e-07
**M2 Macrophage**	CD163	-0.19	1.93e-04	0.297	3.64e-09
	VSIG4	-0.166	1.17e-03	0.167	1.10e-03
	MS4A4A	-0.191	1.85e-04	0.204	6.37e-05
**Neutrophils**	CD66b (CEACAM8)	0.021	6.89e-01	0.199	9.45e-05
	CD11b (ITGAM)	-0.164	1.34e-03	0.183	3.36e-04
	CCR7	-0.292	6.92e-09	0.149	3.66e-03
**Natural killer cell**	KIR2DL1	-0.077	1.37e-01	0.197	1.09e-04
	KIR2DL3	-0.132	1.03e-02	0.119	2.09e-02
	KIR2DL4	-0.165	1.25e-03	0.149	3.55e-03
	KIR3DL1	-0.077	1.37e-01	0.197	1.09e-04
	KIR3DL2	-0.161	1.61e-03	0.187	2.48e-04
	KIR3DL3	-0.02	7.73e-01	0.089	8.34e-02
	KIR2DS4	-0.122	1.76e-02	0.158	2.02e-03
**Dendritic cell**	HLA-DPB1	-0.293	5.78e-09	-0.009	8.59e-01
	HLA-DQB1	-0.282	2.11e-08	-0.002	9.76e-01
	HLA-DRA	-0.276	4.47e-08	0.042	4.20e-01
	HLA-DPA1	0.276	4.32e-08	0.004	9.38e-01
	BDCA-1(CD1C)	-0.285	1.63e-08	0.081	1.17e-01
	BDCA-4(NRP1)	-0.173	7.21e-04	0.45	2.58e-20
	CD11c (ITGAX)	-0.224	1.03e-05	0.231	5.34e-06
**Th1**	T-bet (TBX21)	-0.254	5.25e-07	0.138	7.21e-03
	STAT4	-0.245	1.32e-06	0.293	5.86e-09
	STAT1	-0.104	4.20e-02	0.292	6.80e-09
	IFN-γ (IFNG)	-0.19	1.99e-04	0.125	1.47e-02
	TNF-α (TNF)	-0.281	2.62e-08	0.056	2.75e-01
**Th2**	GATA3	-0.174	6.38e-04	0.15	3.42e-03
	STAT6	0.011	8.36e-01	0.238	2.81e-06
	STAT5A	-0.132	1.01e-02	0.276	4.80e-08
	IL13	-0.002	9.71e-01	-0.031	5.43e-01
**Tfh**	BCL6	-0.135	8.61e-03	0.375	4.24e-14
	IL21	-0.136	8.08e-03	0.124	1.57e-02
**Th17**	STAT3	-0.071	1.65e-01	0.421	1.00e-17
	IL17A	-0.122	1.73e-02	-0.052	3.09e-01
**Treg**	FOXP3	-0.241	1.94e-06	0.084	1.01e-01
	CCR8	-0.168	1.02e-03	0.232	4.89e-06
	STAT5B	-0.023	6.61e-01	0.352	1.70e-12
	TGFβ (TGFB1)	-0.169	9.50e-04	0.234	4.11e-06
**T cell exhaustion**	PD-1 (PDCD1)	-0.175	6.21e-04	0.096	6.24e-02
	CTLA4	-0.197	1.10e-04	0.233	4.38e-06
	LAG3	-0.227	7.57e-06	0.048	3.47e-01
	TIM-3 (HAVCR2)	-0.245	1.35e-06	0.197	1.18e-04
	GZMB	-0.254	5.41e-07	0.077	1.34e-01

Cor: Correlation coefficient; *P*: *P* value; TAM: Tumor-associated macrophages; Th1: T-helper cell 1; Th2: T-helper cell 2; Tfh: Follicular helper T cell; Th17: T-helper cell 17; Treg: Regulatory T cell

## Discussion

The high incidence and mortality rate of GC has driven the search for novel tumor biomarkers and therapeutic targets. However, no universal therapeutic molecular target is currently available. Metabolic remodeling is a distinctive feature of malignant tumors [21]. Internal characteristics of tumor cells and external microenvironmental pressures jointly promote the formation of novel metabolic phenotypes of malignant tumor cells [22]. Glutamine is an important metabolite in tumor occurrence and development [23]. Recent studies have emphasized the important role of glutamine-dependent mechanisms in malignant tumors. Furthermore, inhibitors of glutamine transport and metabolism have been proposed as potential antitumor therapeutics [24, 25]. SLC38A2 is characterized as an important membrane protein that is closely implicated in glutamine transport [7]. It can therefore be hypothesized that SLC38A2 has a distinct role in GC progression.

The results of the present study demonstrated that SLC38A2 was highly expressed in GC tissues, which was associated with a poor prognosis in patients with GC. Subsequently a stable SLC38A2 knockdown cell line was used to perform *in vivo* and *in vitro* experiments to investigate the biological role of SLC38A2 in GC. The results demonstrated that SLC38A2 enhanced GC cell proliferation and metastasis *in vitro*, as well as GC tumor formation *in vivo*. Moreover, SLC38A2 gene mutations, copy number alterations and amplifications were investigated to determine the mechanisms underlying SLC38A2 upregulation in GC tissues. The results demonstrated that amplification, rather than genetic mutations, increased SLC38A2 expression levels. Furthermore, miRNAs and transcription factors are key epigenetic regulators of gene expression [26]. Therefore, miRNAs and transcription factors, which may potentially regulate SLC38A2, were determined using bioinformatics analysis and Cytoscape was used to visualize the SLC38A2 regulatory network. However, additional work is needed to properly characterize the regulatory network of SLC38A2.

The co-expressed gene profiles of SLC38A2 demonstrated that TMUB1 was one of the most negatively associated genes in GC and was first reported by Della Fazia et al. [27]. TMUB1 is a ubiquitin-like protein that translocates from the nucleus to the cytoplasm and is involved in numerous biological processes [28]. A recent study reported that TMUB1 can promote hepatoma cell apoptosis by enhancing the ubiquitination and degradation of the p63 protein [29]. Considering the similarity in localization and function of SLC38A2 and TMUB1, it can be hypothesized that TMUB1 may directly bind and ubiquitinate the SLC38A2 protein. However, the intrinsic relationship between TMUB1 and SLC38A2 still needs further exploration. To understand the biological function of SLC38A2, GO and KEGG enrichment analyses were performed in the present study. The ‘hippo signaling pathway’ was identified as the main biological function and pathway of enrichment. Previous studies have demonstrated the role of the Hippo signaling pathway in tissue regeneration, organ development, stem cell self-renewal and tumorigenesis [30, 31]. Furthermore, the Hippo signaling pathway cascade has been reported to be involved in cancer metabolic reprogramming [32]. To the best of our knowledge the SLC38A2 and Hippo signaling pathway have not previously been reported, and this will therefore be the focus of future work.

Recently, numerous studies have indicated the importance of immune cell infiltration in gastric cancer occurrence and development [32]. There, the role of SLC38A2 in the infiltration of GC immune cells was another focus of the present study. The results demonstrated that the infiltration of CD8 + T cells, macrophages, myeloid dendritic cells, neutrophils and natural killer cells were positively correlated with SLC38A2 expression, suggesting the importance of SLC38A2 in regulating GC tumour immunity. Tumor-associated macrophages (TAMs) are a major tumorigenic immune cell infiltrated in the tumorous environment. Macrophages play a dual role in tumor-bearing hosts: M1 macrophages are generally involved in antitumor immune response, while M2 favorite tumor progression. Accumulating evidence has demonstrated that higher densities of macrophages, especially those with an M2 phenotype, are strongly associated with worse clinical outcomes in a variety of malignancies[33]. Infiltrating TAMs with M2 phenotype are considered as new therapeutic targets for the treatment of malignant tumors[34]. In our study, we found that SLC38A2 expression was positively correlated with M2 macrophage markers, suggesting that high SLC38A2 expression might promote M2 macrophage polarization in GC. However, the specific mechanism by which SLC38A2 regulates M2 polarization has remained elusive, which is the next step in our study. What’s more, emerging as one of breakthroughs for cancer therapy, immunotherapy has become an effective treatment modality after surgery, chemotherapy, radiotherapy, and targeted therapy. Based on all the aforementioned analyses, we hypothesized that SLC38A2 could be a novel target of cancer immunotherapy in GC.

## Conclusion

The present study explored the expression level, clinical value, regulatory factors and biological function of SLC38A2 in GC, as well as its association with immune infiltration. The results demonstrated that SLC38A2 potentially promoted tumor development in GC, including tumor growth, metastasis and immune infiltration. Therefore, these data suggest that SLC38A2 may serve as a potential novel diagnostic and therapeutic target for GC.

## Electronic supplementary material

Below is the link to the electronic supplementary material.

Supplementary Material 1

Supplementary Material 2. Fig. S1. Expression of multiple SLC38A family members in GC tumor tissue in the TCGA database.

Supplementary Material 3. Supplementary Table S1. Primers used in this study.

Supplementary Material 4. Supplementary Table S2. The top 50 positively/negatively associated genes with SLC38A2.

Supplementary Material 5. Fig. S2. Steps of bioinformatics analysis of SLC38A2.

## Data Availability

The datasets generated during and/or analyzed during the current study are available from the corresponding author on reasonable request.

## Abbreviations

SLC38A2: Solute carrier family 38 member 2
GC: gastric cancer
TCGA: the Cancer Genome Atlas
KEGG: Kyoto Encyclopedia of Genes and Genomes
GO: Gene Ontology
TIMER: Tumor Immune Estimation Resource
TAM: tumor-associated macrophage
