# Supplementary figures and images for: Comprehensive analysis of the biological function and immune infiltration of SLC38A2 in gastric cancer
Source: BMC Gastroenterol. 2023 Mar 14;23:74. doi: 10.1186/s12876-023-02689-4 (PMC10015769; doi:10.1186/s12876-023-02689-4)

T4 N4 T5 N5 T6 N6      T1 N1 T2 N2 T3 N3

SLC38A2

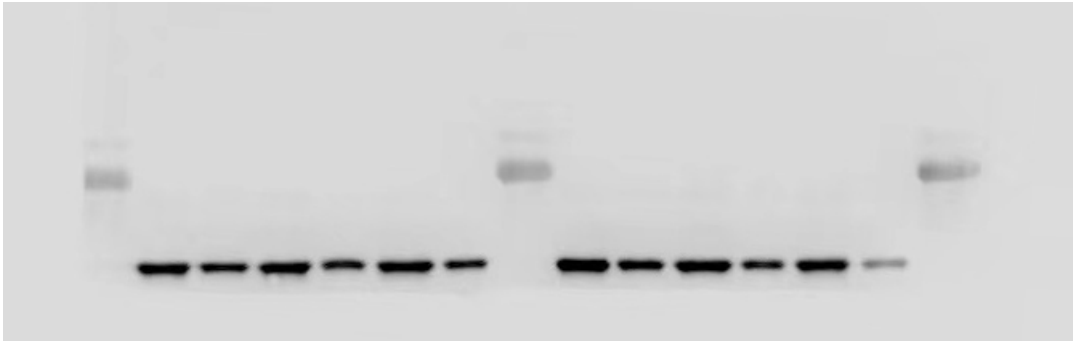

GAPDH

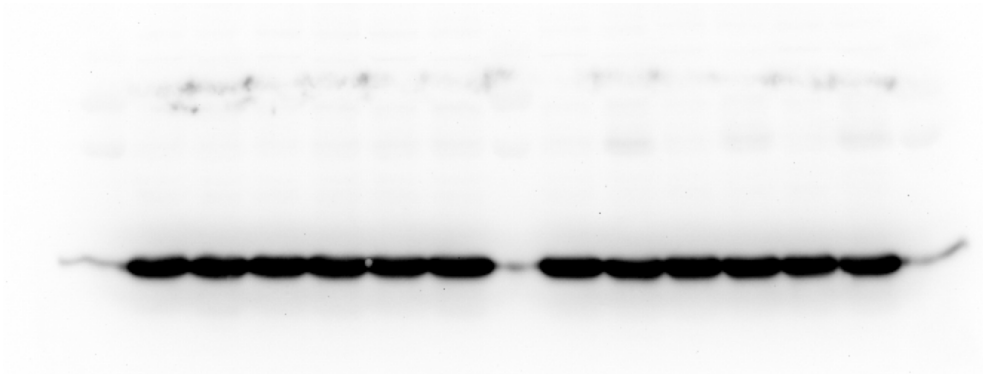

MKN45      AGS      MKN1      HGC27      GES-1

SLC38A2 →

GAPDH →

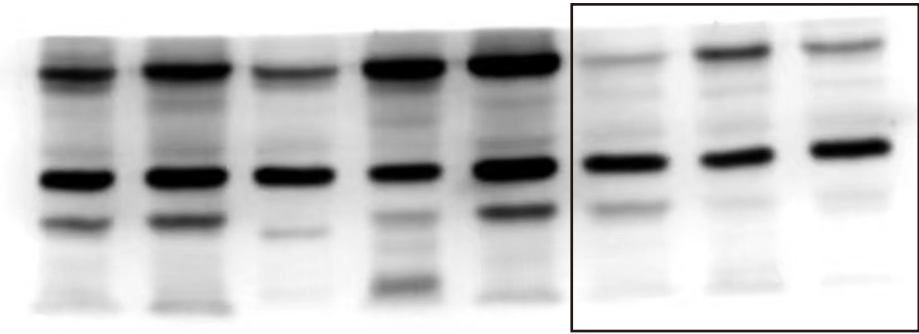

Irrelevant Samples

Supplement: Supplementary file 1 — Supplementary Material 1 [file 12876_2023_2689_MOESM1_ESM.pdf]

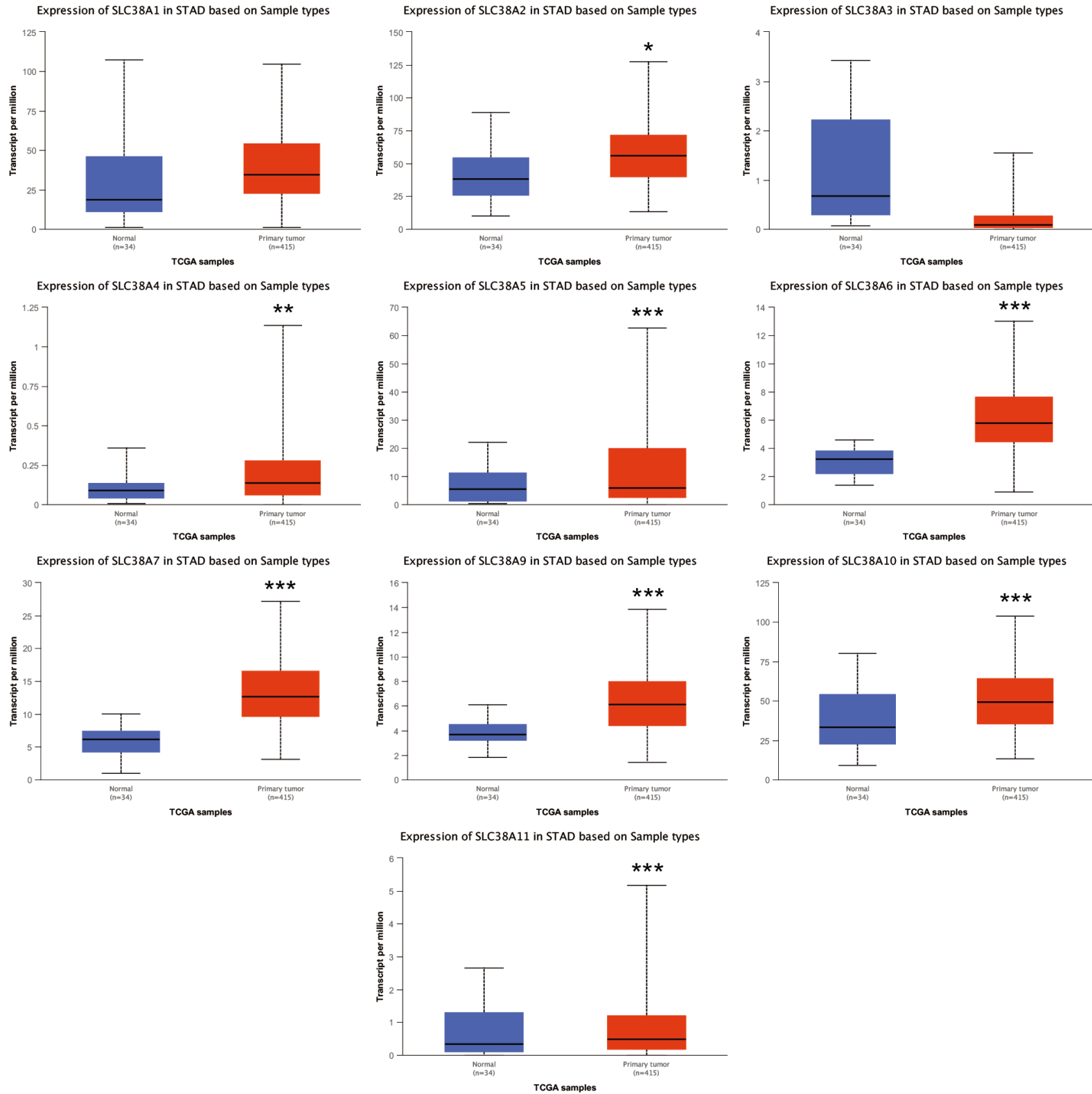

Fig. S1 Expression of multiple SLC38A family members in GC tumor tissue in the TCGA database.

Supplement: Supplementary file 2 — Supplementary Material 2. Fig. S1. Expression of multiple SLC38A family members in GC tumor tissue in the TCGA database. [file 12876_2023_2689_MOESM2_ESM.pdf]

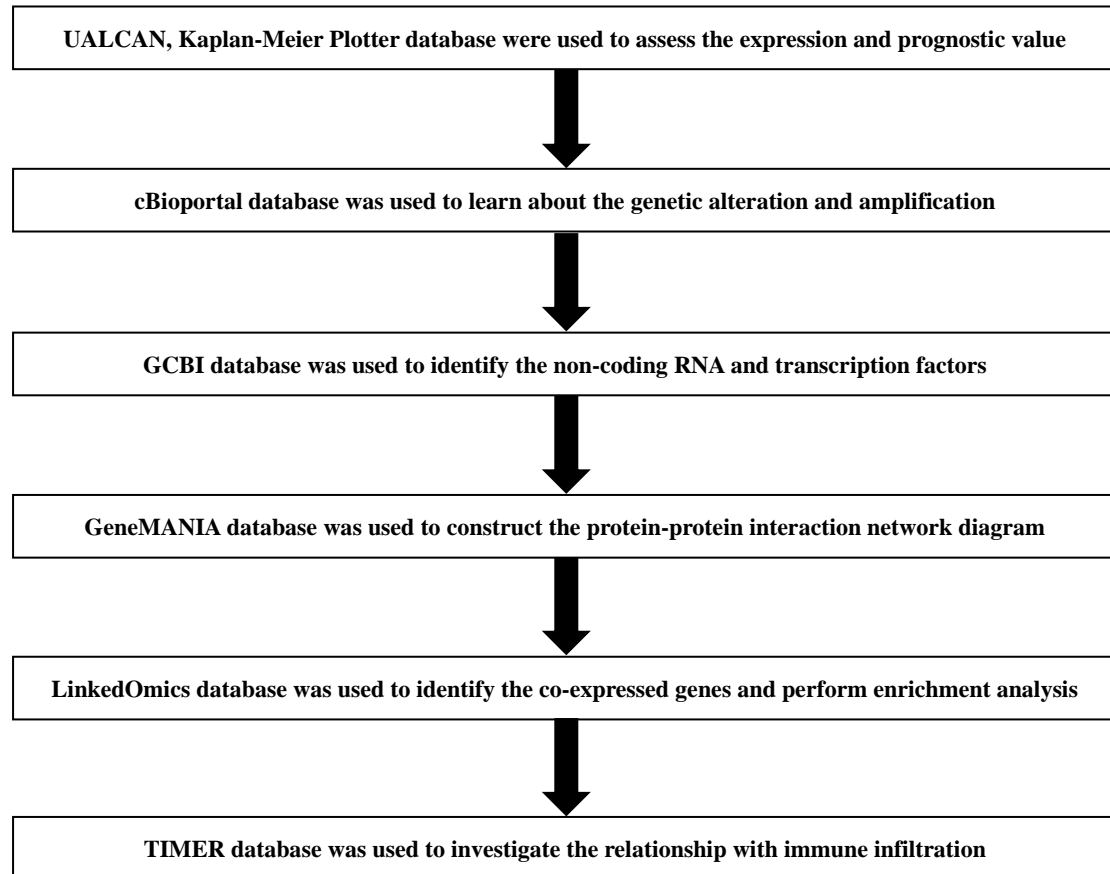

Fig. S2 Steps of bioinformatics analysis of SLC38A2.

Supplement: Supplementary file 5 — Supplementary Material 5. Fig. S2. Steps of bioinformatics analysis of SLC38A2. [file 12876_2023_2689_MOESM5_ESM.pdf]
