# Supplementary material for: Comprehensive analysis of the biological function and immune infiltration of SLC38A2 in gastric cancer
Source: BMC Gastroenterol. 2023 Mar 14;23:74. doi: 10.1186/s12876-023-02689-4 (PMC10015769; doi:10.1186/s12876-023-02689-4)
Supplement: Supplementary file 3 — Supplementary Material 3. Supplementary Table S1. Primers used in this study. [file 12876_2023_2689_MOESM3_ESM.docx]

**Supplementary Table S1. Primers used in this study.**

| Gene | Sequence (5’-3’) |
| --- | --- |
| SLC38A1-F | CCAGTGGCCTAGCTGGTACCAC |
| SLC38A1-R | TCCCCAGCGAAAGTTGACTCAGAC |
| SLC38A2-F | ATGAGTTGCCTTTGGTGATCC |
| SLC38A2-R | ACAGGACACGGAACCTGAAAT |
| SLC38A3-F | ATCTCCAACCTGTCCATCGC |
| SLC38A3-R | GCCAACGGCAATAAGCACAT |
| SLC38A4-F | AGAAATTCCAAATACCCTGCCC |
| SLC38A4-R | GAAGCGTGTTGTTGAATGACAG |
| SLC38A5-F | GTTGGGGCCATGTCCAGTTA |
| SLC38A5-R | AGTGTTTCATGAGGGCGAGG |
| SLC38A6-F | TTGGTGTAGTTGGGTGCCAGT |
| SLC38A6-R | AGCAAAACGAATGCCCCAAG |
| SLC38A7-F | GAAAGAATCCCCCAAGCTCCA |
| SLC38A7-R | TTGGGCTACTTCTGTGCTGG |
| SLC38A8-F | TGCGTGAGTCCCATCCTTC |
| SLC38A8-R | GCAGCTTCGTGACACTGAAAC |
| SLC38A9-F | CCTGTTTGGACTAGGAGCCC |
| SLC38A9-R | ACCCTCAGTTTCCTTCTCTGAGC |
| SLC38A10-F | CTCATGGATGACGCACCAGT |
| SLC38A10-R | CGTGGAATGCCAGGCCG |
| SLC38A11-F | CAGCATCTTCCCACCTGCTGA |
| SLC38A11-R | ATAAGGCAATCCTGCGGCG |
| GAPDH-F | ACAACTTTGGTATCGTGGAAGG |
| GAPDH-R | GCCATCACGCCACAGTTTC |
