# Supplementary material for: Comprehensive analysis of the biological function and immune infiltration of SLC38A2 in gastric cancer
Source: BMC Gastroenterol. 2023 Mar 14;23:74. doi: 10.1186/s12876-023-02689-4 (PMC10015769; doi:10.1186/s12876-023-02689-4)
Supplement: Supplementary file 4 — Supplementary Material 4. Supplementary Table S2. The top 50 positively/negatively associated genes with SLC38A2. [file 12876_2023_2689_MOESM4_ESM.docx]

**Supplementary Table S2. The top 50 positively/negatively associated genes with SLC38A2.**

| **Posetive genes** | **Statistic** | ***P*-value** | **FDR** | **Negative genes** | **Statistic** | ***P*-value** | **FDR** |
| --- | --- | --- | --- | --- | --- | --- | --- |
| SLC38A2 | 1 | 1.00E-35 | 1.00E-31 | C6orf108 | -0.4576466 | 7.13E-23 | 7.21E-20 |
| OSBPL8 | 0.5581626 | 2.32E-35 | 2.35E-31 | FAM195A | -0.455033 | 1.33E-22 | 1.29E-19 |
| TRAPPC6B | 0.53010048 | 1.91E-31 | 1.29E-27 | TMUB1 | -0.4432573 | 2.11E-21 | 1.30E-18 |
| CDK17 | 0.5176968 | 7.89E-30 | 3.99E-26 | PEX16 | -0.435952 | 1.11E-20 | 5.23E-18 |
| SFRS2IP | 0.5038079 | 4.27E-28 | 1.62E-24 | CISD3 | -0.4358403 | 1.14E-20 | 5.24E-18 |
| WDR47 | 0.50338532 | 4.81E-28 | 1.62E-24 | CLN6 | -0.4329094 | 2.19E-20 | 9.65E-18 |
| MAP4K5 | 0.50270717 | 5.81E-28 | 1.68E-24 | IMP4 | -0.4313949 | 3.07E-20 | 1.24E-17 |
| AFF4 | 0.48934452 | 2.25E-26 | 5.70E-23 | MRM1 | -0.4276768 | 6.95E-20 | 2.56E-17 |
| USP15 | 0.4754548 | 8.54E-25 | 1.92E-21 | C2orf7 | -0.4257583 | 1.06E-19 | 3.60E-17 |
| RICTOR | 0.47428345 | 1.15E-24 | 2.33E-21 | PLEKHJ1 | -0.4248995 | 1.27E-19 | 4.22E-17 |
| SOCS4 | 0.47290677 | 1.63E-24 | 3.00E-21 | GFER | -0.4242821 | 1.45E-19 | 4.74E-17 |
| TNKS2 | 0.47194296 | 2.09E-24 | 3.52E-21 | RALY | -0.4211024 | 2.88E-19 | 8.64E-17 |
| KIAA1033 | 0.46837738 | 5.11E-24 | 7.92E-21 | NUBP2 | -0.4208934 | 3.01E-19 | 8.82E-17 |
| EXOC5 | 0.46809889 | 5.48E-24 | 7.92E-21 | HDHD3 | -0.4204206 | 3.33E-19 | 9.37E-17 |
| TMTC3 | 0.4669911 | 7.22E-24 | 9.74E-21 | FAM173A | -0.4204087 | 3.34E-19 | 9.37E-17 |
| ZFYVE16 | 0.4646762 | 1.28E-23 | 1.62E-20 | CHCHD10 | -0.4196964 | 3.88E-19 | 1.08E-16 |
| BNIP2 | 0.46383669 | 1.58E-23 | 1.88E-20 | SLC25A1 | -0.4195541 | 4.00E-19 | 1.09E-16 |
| MFAP3 | 0.46165427 | 2.69E-23 | 3.03E-20 | TPRN | -0.4164048 | 7.79E-19 | 1.88E-16 |
| RC3H2 | 0.46020649 | 3.83E-23 | 4.08E-20 | HDAC10 | -0.4157097 | 9.02E-19 | 2.12E-16 |
| CAMSAP1L1 | 0.45377274 | 1.80E-22 | 1.66E-19 | BRP44 | -0.4134465 | 1.45E-18 | 3.33E-16 |
| LOC100132724 | 0.45266673 | 2.35E-22 | 2.06E-19 | PMF1 | -0.4117145 | 2.07E-18 | 4.56E-16 |
| SECISBP2L | 0.451947 | 2.78E-22 | 2.34E-19 | ACTR1B | -0.4110154 | 2.40E-18 | 4.96E-16 |
| ARID2 | 0.45044035 | 3.97E-22 | 3.21E-19 | TMEM177 | -0.4108684 | 2.47E-18 | 5.05E-16 |
| CAPRIN2 | 0.44988691 | 4.52E-22 | 3.52E-19 | RPIA | -0.4098683 | 3.04E-18 | 5.90E-16 |
| RLF | 0.44857643 | 6.15E-22 | 4.61E-19 | DECR2 | -0.4088489 | 3.74E-18 | 7.21E-16 |
| TCP11L2 | 0.44794361 | 7.13E-22 | 4.99E-19 | DHRS11 | -0.4082336 | 4.25E-18 | 8.03E-16 |
| BACH1 | 0.44792641 | 7.16E-22 | 4.99E-19 | YDJC | -0.4074878 | 4.95E-18 | 9.18E-16 |
| NFAT5 | 0.44751533 | 7.88E-22 | 5.31E-19 | BIN1 | -0.4057129 | 7.10E-18 | 1.29E-15 |
| FOXO1 | 0.44409864 | 1.74E-21 | 1.13E-18 | DCI | -0.4053588 | 7.63E-18 | 1.38E-15 |
| FAM160B1 | 0.44399707 | 1.78E-21 | 1.13E-18 | MPND | -0.4043487 | 9.36E-18 | 1.65E-15 |
| PPFIBP1 | 0.44303614 | 2.22E-21 | 1.32E-18 | GHDC | -0.4040613 | 9.92E-18 | 1.71E-15 |
| TBC1D15 | 0.44217628 | 2.71E-21 | 1.57E-18 | ZNF787 | -0.4019602 | 1.51E-17 | 2.43E-15 |
| MON2 | 0.4415711 | 3.11E-21 | 1.72E-18 | DUS1L | -0.401306 | 1.73E-17 | 2.68E-15 |
| ZDHHC17 | 0.44152634 | 3.14E-21 | 1.72E-18 | TOR3A | -0.4008164 | 1.90E-17 | 2.89E-15 |
| PAFAH1B1 | 0.43973848 | 4.73E-21 | 2.52E-18 | APOA1BP | -0.3994294 | 2.51E-17 | 3.70E-15 |
| PIK3CA | 0.43786257 | 7.23E-21 | 3.75E-18 | DNPEP | -0.3991311 | 2.66E-17 | 3.87E-15 |
| KLHL28 | 0.43729259 | 8.22E-21 | 4.16E-18 | TEX264 | -0.3985729 | 2.97E-17 | 4.20E-15 |
| TRIM23 | 0.43708182 | 8.62E-21 | 4.25E-18 | MLST8 | -0.3979848 | 3.34E-17 | 4.66E-15 |
| CLIC4 | 0.43640962 | 1.00E-20 | 4.83E-18 | C1orf35 | -0.3972845 | 3.83E-17 | 5.14E-15 |
| CLIP1 | 0.43449356 | 1.54E-20 | 6.93E-18 | ZNF768 | -0.3962123 | 4.74E-17 | 6.26E-15 |
| DNAJB4 | 0.43199762 | 2.69E-20 | 1.16E-17 | TOR2A | -0.3959511 | 4.99E-17 | 6.55E-15 |
| ARHGAP29 | 0.43151659 | 2.99E-20 | 1.23E-17 | NUDT14 | -0.3954502 | 5.50E-17 | 7.09E-15 |
| ZNF644 | 0.43151577 | 2.99E-20 | 1.23E-17 | GALM | -0.395341 | 5.62E-17 | 7.15E-15 |
| CEP170 | 0.4306154 | 3.65E-20 | 1.45E-17 | RAB20 | -0.3952736 | 5.69E-17 | 7.20E-15 |
| NIN | 0.4284296 | 5.90E-20 | 2.29E-17 | SSNA1 | -0.3952093 | 5.77E-17 | 7.24E-15 |
| JMJD1C | 0.427822 | 6.73E-20 | 2.56E-17 | SIRT6 | -0.3947681 | 6.29E-17 | 7.71E-15 |
| LYST | 0.4277147 | 6.89E-20 | 2.56E-17 | MRPS34 | -0.3938841 | 7.47E-17 | 8.86E-15 |
| LRRC8C | 0.42735483 | 7.46E-20 | 2.69E-17 | RPP25 | -0.3938137 | 7.57E-17 | 8.86E-15 |
| SLK | 0.42645466 | 9.07E-20 | 3.22E-17 | THAP7 | -0.3933708 | 8.26E-17 | 9.60E-15 |
| RANBP9 | 0.42606346 | 9.88E-20 | 3.44E-17 | C2orf24 | -0.3931238 | 8.66E-17 | 9.96E-15 |

Abbreviation: FDR, False Discovery Rate.
